# Health Symptoms and Proximity to Active Multi-Well Unconventional Oil and Gas Development Sites in the City and County of Broomfield, Colorado

**DOI:** 10.3390/ijerph20032634

**Published:** 2023-02-01

**Authors:** Meagan L. Weisner, William B. Allshouse, Benjamin W. Erjavac, Andrew P. Valdez, Jason L. Vahling, Lisa M. McKenzie

**Affiliations:** 1 Department of Public Health and Environment, City and County of Broomfield, Broomfield, CO 80020, USA; 2 Department of Environmental & Occupational Health, University of Colorado Anschutz Medical Campus, Aurora, CO 80045, USA; 3 Department of Strategic Initiatives, City and County of Broomfield, Broomfield, CO 80020, USA

**Keywords:** epidemiology, unconventional oil and gas development, health symptoms, air pollution, hydraulic fracturing, acute exposure symptoms

## Abstract

City and County of Broomfield (CCOB) residents reported over 500 health concerns between January 2020 and December 2021. Our objective was to determine if CCOB residents living within 1 mile of multi-well unconventional oil and gas development (UOGD) sites reported more frequent health symptoms than residents living > 2 miles away. We invited 3993 randomly selected households to participate in a health survey. We applied linear regression to test associations between distance to UOGD and summed Likert scores for health symptom categories. After covariate adjustment, respondents living within 1 mile of one of CCOB’s UOGD sites tended to report higher frequencies of upper respiratory, lower respiratory, gastrointestinal and acute symptoms than respondents living more than 2 miles from the sites, with the largest differences for upper respiratory and acute symptoms. For upper respiratory and acute symptoms, scores differed by 0.81 (95% CI: 0.06, 2.58) and 0.75 (95% CI: 0.004, 1.99), respectively. Scores for adults most concerned about air pollution, noise and odors trended higher within 1 mile for all symptom categories, while scores among adults least concerned trended lower. Scores trended higher for lower respiratory, gastrointestinal and acute symptoms in children living within 2 miles of UOGD, after covariate adjustment. We did not observe any difference in the frequency of symptoms reported in unadjusted results. Additional study is necessary to understand relationships between proximity to UOGD and health symptoms.

## 1. Introduction

### 1.1. Unconventional Oil and Gas Development

The United States (US) is now the world’s top producer of both oil and natural gas [1], largely because of advances in extraction technology over the past 20 years [2,3]. These technological advances allow operators to co-locate many wells on one site (multi-well sites) and reduce the number of well pads in an area, as well as pipeline routes, and production facilities [4]. However, there is a growing concern regarding the increase in intensity, frequency and duration of air pollutant and noise emissions related to multi-well unconventional oil and gas development (hereinafter referred to as UOGD) sites [5,6].

Colorado is among the top five oil-producing states, with the majority of UOGD sites operating within the Denver–Julesburg basin (DJB) in northeast Colorado, including the urban corridor along the Northern Front Range [7]. Concurrent to UOGD growth, the Northern Front Range has experienced intensive population growth over the past 20 years [8]. Co-current intensive population and UOGD growth in the DJB led to a 14% increase in the size of the DJB population living within 1 mile of an UOGD site between 2000 and 2012 [9]. The rate of population growth continues to increase across Colorado’s Front Range, but especially within the City and County of Broomfield (CCOB). The CCOB is expected to experience a population increase of 24% by 2030 (from 2020 population counts) [10], double the projected population increase of 12% for the entire state [8]. Approximately 10.4% of CCOB’s 74,112 residents currently live within 1 mile of 6 multi-well UOGD sites; another 15.1% live between 1 and 2 miles from the sites [11].

### 1.2. Oil and Gas History in the City and County of Broomfield

The CCOB is one of two consolidated municipal and county governments in the state of Colorado [12], created out of parts of four neighboring counties in 2001. These adjacent counties are as politically and economically diverse as Boulder County, which has historically had a progressive environmental and conservation ethos [13], and Weld County, whose economy relies heavily on resource and mineral extraction [14]. This diversity provides a unique nexus and test case for the risks, challenges and opportunities relating to UOGD in proximity to urban environments.

In 2018, a Denver-based UOGD operator received permits [15] from the Colorado Oil and Gas Conservation Commission (COGCC) to drill 84 unconventional oil and gas wells across six UOGD sites in the rapidly urbanizing area of north/central Broomfield. Prior to COGCC approval and amidst much public outcry from high-income neighborhoods opposing the construction of the UOGD sites, city officials negotiated the final locations of the pads along with an agreement that enumerated a series of measures (referred to as best management practices, or BMPs) intended to reduce impacts to health, safety and the environment [16]. 

**Figure 1:**
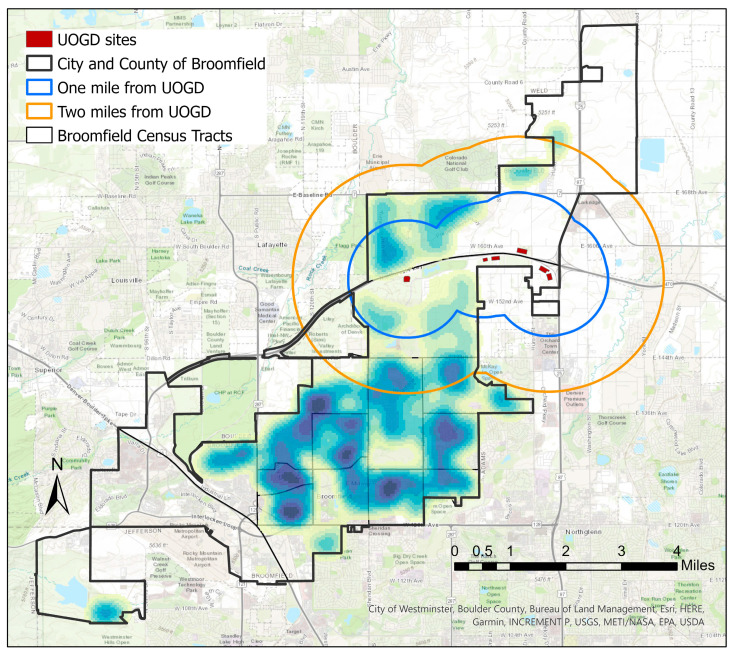
Kernel Density map indicating location densities of 3993 households randomly selected to participate in the health survey. Darker colors indicate a greater number of surveys were sent to that location.

The final, negotiated well pad locations were constructed on CCOB-owned public lands and are surrounded by single-family residential areas, some of which are 1000 ft. from the nearest pad (see UOGD locations in Figure 1). After the approval of the locations for the six UOGD sites, strong opposition from nearby residents remained, and concerns were raised about cumulative exposures to toxic air emissions from living near multiple sites, as well as exposure to additional traffic, dust, noise and lighting. One year after the six multi-well pad project was approved, Colorado Senate Bill 181 (SB-181), which paved the way for the COGCC to adopt new oil and gas locations setback distances at a minimum of 2000 ft. from occupied residential structures, was passed by the state legislature. This landmark bill was supported, in part, by a risk assessment that demonstrated a potential for health impacts to occur up to 2000 ft. from UOGD as a result of possible exposure to air toxics [17,18]. 

### 1.3. Air and Noise Pollution 

Multiple studies have demonstrated potential impacts to human health from air pollutants emitted from Colorado’s UOGD well sites [5,6,18,19]. The CCOB has a robust air quality monitoring network with 14 sensors surrounding the six multi-well UOGD sites [20]. Previous studies indicate that the use of BMPs, such as closed loop flowback systems, can reduce the frequency of increased ambient air volatile organic compounds (VOCs) concentrations [21]. 

While BMPs, such as the closed loop flowback systems used in Broomfield, lower air pollutant emissions from UOGD well sites, reducing VOCs during pre-production activities is dependent upon available technological mitigations. Broomfield’s monitoring system has demonstrated that, regardless of strict BMPs, there are still numerous increases in the frequency, magnitude and duration of VOC emissions during pre-production operations, especially during well bore drilling (likely attributed to drill cuttings containing hydrocarbons) and coiled tubing/mill out [22]. 

Noise from UOGD occurs in nearly all phases of well development and into production [6,23]. Noise can disturb nearby residents and disproportionately impact vulnerable populations, including the elderly and chronically ill [24]. Recent studies indicate that BMPs, such as sound walls, are not effective in mitigating noise exposures for nearby residents [6,25]. 

### 1.4. Human Health Impacts and Proximity to UOGD

Several months after the commencement of construction and drilling at several of the UOGD sites, residents began to complain to city officials of health symptoms they believed were caused by air toxics and noise related to UOGD [26]. The CCOB’s Department of Public Health and Environment formalized a way for residents to submit health complaints online and within a 2-year period (2020–2021), during which time several well pads were being constructed, residents reported over 500 health concerns. The majority of concerns were related to air pollution, noise and odor associated with six multi-well UOGD sites [26]. Health concern reports increased during pre-production and included reports of headaches, eye and throat irritation, and nosebleeds. Over all phases of UOGD, residents most commonly reported difficulty sleeping and anxiety or stress and often stated noise disturbances from nearby oil and gas operations as the cause [27]. The Human Health Risk Assessment [17,18], which gave support for the passage of Colorado Senate Bill 181 (SB-181), was the basis for CCOB’s health collection efforts, as the risk assessment recommended efforts focusing on population-specific, local data collection.

The body of epidemiological literature indicates that UOGD affects the health of nearby residents. A current review found that, in 25 of 29 studies, there was at least one statistically significant association between UOGD exposure and adverse health outcomes (hospitalizations, adverse birth outcomes, cancer and asthma exacerbations) [28]. More recently published studies report associations between intensity of oil and gas activity and indicators and exacerbations of cardiovascular disease [29,30,31], and further evidence of associations with adverse birth outcomes [32,33,34]. Additionally, residents living within 1 km of an UOGD site self-report more skin conditions and upper respiratory symptoms than those living farther away [35]. 

Several studies document sociopsychological impacts in residents living near UOGD sites due to anxieties related to the potential release of toxins and carcinogens [36]. Commonly reported symptoms from those living near UOGD included psychosocial stress associated with community change [23], worry [37,38] and adverse mental [39] and physical health effects [40]. As UOGD outpaces the scientific community’s ability to understand potential health effects, studies of self-reported outcomes are a vital way to understand health impacts in order to influence public policy [40]. After the onset of pre-production UOGD activities and the notably large number of symptoms reported to CCOB’s Department of Public Health and Environment, it became clear that a more robust study was needed to better assess self-reported health symptoms and distance to UOGD. Our objective is to determine whether CCOB residents living near the CCOB’s multi-well oil and gas sites, which are considered to have some of the most rigorous BMPs in the State of Colorado, report more health symptoms than CCOB residents not living near the sites. At the time this study was conducted, no other studies have aimed to associate symptoms at various proximities to UOGD in a jurisdiction that requires such extensive BMPs. 

## 2. Materials and Methods

We conducted a cross-sectional study of 3993 randomly selected CCOB households to collect data on self-reported health symptoms between October and December 2021. 

### 2.1. Study Area and Population

The CCOB is located in Colorado’s Front Range with a total land area of 33 square miles and a population of approximately 74,000 [11]. In 2021, CCOB was rated as the fifth healthiest county in the United States, according to research conducted by University of Missouri Extension Center for Applied Research and Engagement Systems (CARES) [41]. Seventy-six percent of the population identifies as white alone [11]; 59% have obtained a Bachelor’s degree or higher; and the median household income is $107,638, nearly one- third higher than the state of Colorado’s median household income [42]. Prior to the start of this research, 84 UOGD wells were permitted for development in Northern CCOB (Figure 1) located across six sites. During the time health surveys were being collected, 30 wells were in the production phase, with another 21 wells in the pre-production phase (drilling, hydraulic fracturing and/or the coiled tubing/mill out), and 33 wells had no activity.

### 2.2. Survey Instrument

We designed our survey and questions based on symptoms collected in prior oil and gas survey research [35,43] as well as symptoms collected by the State of Colorado’s Department of Public Health and Environment’s Oil and Gas Health Information and Response line, and the CCOB’s Health Concern line. An important objective of this research is to understand symptoms and proximity to UOGD sites, and building off symptoms defined in the previous literature helps to characterize how the population in CCOB may report similar or different symptoms related to living near or away from UOGD. Surveys contained Likert scale questions, commonly used in epidemiological survey research [35,43], and provided a way to quantify responses. We asked about the frequency of 20 separate symptoms experienced in the past 14 days. Choices included never, once, 2–5 times, 5–13 times or everyday (0–4 Likert scale). The survey also contained questions on occurrence of each symptom (yes, no) within the past two and five years: before major UOGD projects began, preexisting chronic health conditions, demographics, household size, smoking (tobacco and marijuana) status and exercise habits, as well as the degree of concern for nine environmental issues (e.g., noise, odor, air, etc.) by using a 0–4 Likert scale—not at all concerned, slightly, somewhat, moderately or extremely concerned (see Supplemental Material, “Survey”). Survey data were collected using ESRI’s ArcGIS Survey123 platform.

### 2.3. Household Selection and Recruitment 

Households were randomly selected using ArcGIS Desktop version 10.8.1. Random selection helped reduce participant bias and ensure households were targeted at locations throughout CCOB. There are approximately 21,000 residential parcels in CCOB [11], and 19% received a postcard in the mail with a survey link. Approximately one-fourth of the total number of parcels are located in CCOB’s two northernmost census tracts; these tracts contain all UOGD activity in CCOB. Since the survey mentioned that this research was related to UOGD activity, we expected a greater response rate from those living in the census tracts near UOGD than from those living in the tracts farther away. To ensure an adequate sample size was collected, we weighted the distribution of randomly selected households for population density while also oversampling in the southernmost census tracts (which are located further from UOGD) (Figure 1). To accomplish this, a fishnet grid was created for the two northernmost census tracts and again for the 13 southernmost census tracts. The centroid of each grid was attached to the nearest residential parcel for household selection. Initially, about 1800 households were selected throughout CCOB to receive surveys in the mail. However, due to a low response rate within the first month of data collection, this process was repeated again and approximately 2200 additional households were selected to receive postcards with a link to the survey. Households were also sent two to three reminder postcards to encourage participation. Overall, we sent postcards with a link to the survey via QR code to 524 households within 1 mile of UOGD, 693 households within 1–2 miles of UOGD and 2776 households located >2 miles from UOGD activity.

Postcards were translated into English and Spanish and stated the intent of the research, the length of time expected to complete the health survey (20–30 min) and instructions for accessing the survey via QR code or webpage (see Supplemental Material, “Postcard”). Survey questions were available in English and Spanish.

We asked that only one adult complete the survey per household. To reduce selection bias, instructions asked that the adult selected was the one whose birthday was closest to the date they received the survey in the mail and that the adult lives in the household full time. We encouraged additional survey questions for children to be completed per household by an adult for a child under the age of 18 living in their household, if applicable, and asked that the same selection method be applied for the child. The Institutional Review Board for the University of Colorado (COMIRB) reviewed and approved this research (COMIRB# 21-3719).

### 2.4. Residence Proximity to Nearest Multi-Well Oil and Gas Site

We used ArcGIS to calculate the distance between each respondent’s residence and each UOGD site in CCOB. We then classified residences according to their distance from the nearest UOGD site into distance bands of less than 1 mile, 1–2 miles or >2 miles. We based the 1- and 2-mile cut points on residential locations for CCOB residents filing health complaints attributed to UOGD between 2019 and 2021 [26]. Complainants lived predominantly in CCOB’s two northernmost census tracts where homes are within 2 miles of the multi-well oil and gas sites, with most complainants living within 1 mile of a multi-well oil and gas site.

### 2.5. Outcomes

We grouped health symptoms from the household survey based on physiological organ system [35] and mental health. We assigned: coughs, nasal congestion, runny nose, throat irritation and bloody noses to upper respiratory; shortness of breath and lung irritation to lower respiratory; dizziness, difficulty concentrating, headaches, numbness and tingling, ringing ears and hearing loss, and muscle aches and weakness to neurological; nausea and vomiting to gastrointestinal; and anxiety and stress, difficulty sleeping, difficulty concentrating, and lack of energy and fatigue to mental health. 

We also performed a principal component analysis on all symptoms. The first three components capture 58% of the variability, with Eigenvalues > 1. The first component captured 33% of the variability, with Eigenvectors > 0.1 for all symptoms. The second component captured 7.2% of the variance, with Eigenvectors > 0.1 for primarily mental health symptoms (anxiety/stress, difficulty concentrating, lack of energy/fatigue, difficulty sleeping). The third component captured another 6.6% of the variance, with Eigenvectors > 0.1 for several acute symptoms (nausea, vomiting, nosebleeds, lung irritation, shortness of breath, cough and throat irritation). Based on these PCA results, we assigned all symptoms to an outcome group named total symptoms and symptoms with Eigenvectors > 0.1 in the third component to an outcome group named acute response. We had already created a mental health outcome group with the symptoms loading to the second component.

### 2.6. Statistical Analysis

The mean Likert score for each self-reported outcome and the total number of symptoms reported as occurring at least once (Likert score > 0) in the past 14 days for each respondent were calculated according to the distance of the respondent’s residence (<1, 1–2 or >2 miles) from the nearest multi-well UOGD site. Because many symptoms surveyed may also be associated with COVID and of higher COVID infection rates in Colorado’s Hispanic/Latino, Native Hawaiian/Pacific Islander, American Indian/Alaska Native and African American communities in 2021 [44], we assigned responses from the race/ethnicity choices into two groups: (1) White, Asian, Asian/White or Asian/White/Native Hawaiian/other Pacific Islander and (2) Hispanic/Latino, Hispanic/Latino/White, American Indian/Alaska Native, Native Hawaiian/other Pacific Islander or Black/White, or other. No survey respondents identified as only Black. We classified reported occupation as management or professional; service, sales, or office; natural resources, construction, maintenance, production, transportation, or material moving; and not working [45].

Because the distribution of the summed Likert scores for adults was log normal, all summed scores were log transformed to approximate a normal distribution. Summed Likert scores for children approximated a normal distribution. We applied the method of least squares linear regression to test the association between residence distance from the closest UOGD site (distance band) and the mean overall number of symptoms and mean summed Likert scores for all symptoms as well as the mean summed Likert scores for each of six groups of health symptoms (upper respiratory, lower respiratory, gastrointestinal, neurological, acute response and mental health, see supplemental material, Table S1) for unadjusted and adjusted models. We ran separate models for adult respondents and children (<18 years) because children did not provide their own responses and there were fewer covariates available for children. Based on a priori knowledge of their association with both exposure and outcomes, we adjusted both the adult and child models for age (ordinal), gender identification (male and female or other), smoked or smoker ever present in household (yes/no), number of chronic health conditions reported (continuous) and number of children under 18 years of age living in household (continuous). The adult model was also adjusted for days per week of exercise (continuous), alcoholic drinks consumed each week (ordinal), hours per day spent at residence (ordinal), level of education (ordinal), race/ethnicity (dichotomous), occupation (categorical) and years at current residence (<, ≥2 years). An evaluation of correlation between co-variates indicated little correlation between covariates. We evaluated for effect modification by performing stratified analysis for gender, smoker ever present in household, years at current residence, age (<, ≥55 years), number of chronic conditions (0, >0) and hours per day spent at residence (<, ≥13 h). Additionally, we evaluated for mediation as well as effect modification by the three most frequently reported environmental concerns (air pollution, noise and odors) in CCOB’s oil and gas complaint database [26] by using multiple linear regression with (1) the mediator (sum of Likert scores for air pollution, noise and odor concerns) as the dependent variable and setback distance as the main predictor; (2) the sum of Likert scores for a group of health symptoms as the dependent variable and the mediator as the main predictor; and (3) the sum of Likert scores for a group of health symptoms as the dependent variable and setback distance as the main predictor [46]. We considered mediation to be present if all three regressions returned statistically significant results for the main predictor [47]. To evaluate for effect modification, we stratified by the median summed Likert score (<, ≥4) for air pollution, noise and odor concerns, as well as stratifying by the median summed Likert score (<, ≥8) for the remaining seven environmental concerns in the survey (light, dust, wildlife, traffic, water, oil spill, waste). Given the exploratory nature of this study, no adjustments were made for multiple comparisons, and significance was established at the two-sided 0.05 level. We conducted all statistical analysis using SAS 9.3 (SAS Institute, Cary, NC, USA).

## 3. Results

Four hundred twenty-seven adults responded to our survey, and 59 adults provided responses for a child living in their household. Response rates for the three distance bands ranged from 10–11.6%, and the overall response rate was 10.7% (Table 1).

### 3.1. Demographics

Demographic results are presented in Table 1. According to the U.S. Census [11], CCOB does not have extensive racial diversity, with 76% of respondents identifying as white alone, which is reflected in our survey respondents. In general, a higher proportion of respondents living within 1 mile and 1–2 miles (than from those living more than 2 miles away) from one of CCOB’s UOGD sites identified as male, never smoked or lived with someone that smoked, consumed more alcoholic beverages, were aged 55 years or older, spent less time at home each day and lived less than 2 years in their current home. Level of concern with oil and gas stressors (air pollution, noise and odors) did not differ by distance band. 

### 3.2. Self-Reported Health Symptoms

We observed no differences in unadjusted analysis of self-reported health symptoms by setback distance (Table 2). A full list of symptoms can be viewed in Supplemental Material (Table S1).

After covariate adjustment, the total number of symptoms reported at least once in the past 14 days and summed Likert scores for all symptoms trended higher as distance to UOGD decreased (Table 3). Respondents living within 1 mile of one of CCOB’s UOGD sites tended to report higher frequencies of upper respiratory, lower respiratory, gastrointestinal and acute symptoms than respondents living 1–2 miles and more than 2 miles from the sites, with the largest differences for upper respiratory and acute symptoms. Mean summed Likert scores differed by 0.81 (95% CI: 0.06, 2.58) and 0.75 (95% CI: 0.004, 1.99) for upper respiratory and acute symptoms, respectively. We observed null results for mental health and neurological symptoms in our adjusted model.

Level of concern for the top three environmental complaints (noise, odor and air) in CCOB’s oil and gas complaint database did not mediate the relationships between reported symptoms and distance band (see Supplemental Material, Tables S2 and S3); however, air pollution, noise and odor concerns did modify the relationship (Table 3). Among respondents reporting greater concern for air pollution, odor and noise (sum Likert scores within the top 50th percentile, ≥4), those living within 1 mile of CCOB’s UOGD sites reported 2.88 more health symptoms in the past 14 days (95% CI: 1.14, 4.63) than those living > 2 miles from the sites. Among these respondents, we also observed a 7.26 mean difference in the sum of Likert scores for the sum of all symptoms (95% CI: 3.16, 11.35) and statistically higher means for all symptom categories between those living <1 mile and >2 miles from the UOGD sites (Table 4). Among respondents reporting less concern for air, odor and noise (Sum Likert scores less than the 50th percentile, <4), those living within 1 mile of CCOB’s UOGD sites reported fewer health symptoms in the past 14 days and lower frequencies of all symptom categories than those living >2 miles. Stratified analysis by the median summed Likert score (<, ≥8) for the six remaining environmental concerns indicate effect modification to a lesser extent (see Supplemental Material Table S4).

While we did not observe modification by gender identification, we did observe greater mean differences in summed Likert scores for upper respiratory (1.54, 95%CI: 0.16, 2.97) and acute symptoms (1.30, 95%CI: 0.13, 2.47) in respondents identifying as male. Likewise, while we did not observe modification by number of chronic conditions, we did observe lower mean differences in summed Likert scores for upper respiratory (0.75, 95%CI:−0.83, 2.33) and greater differences for acute symptoms (1.20 95%CI: −0.16, 2.58) in respondents reporting more than one chronic condition (see Supplemental Material, Table S5). In sensitivity analyses for respondents that identified as white, never smoked or lived with someone that smoked, lived in their current home for 2 or more years, were at home more than 12 h per day and were aged 55 years or older, we observed results similar to results for all respondents (see Supplemental Material, Table S6).

### 3.3. Results for Children

Fifty-nine respondents reported health symptoms for one child in their household. Because of the small population of children, we compared children living < 2 miles to children living > 2 miles from Broomfield’s UOGD sites. Parents living < 2 miles from a Broomfield UOGD site reported more symptoms and higher frequencies of all symptoms, except neurological symptoms, in their children than those living more than 2 miles from the sites, after covariate adjustment (Table 4). The mean total number of symptoms differed by 2.29 (95% CI: 0.05, 4.53), and mean summed Likert scores differed by 0.83 (95% CI: 0.12, 1.54), 0.81 (95% CI: 0.3, 1.31) and 2.38 (95% CI: 0.36, 4.41) for lower respiratory, GI and acute symptoms, respectively, between children residing <2 and >2 miles from Broomfield’s UOGD sites. We observed null results for mental health and neurological symptoms in our adjusted model for children.

## 4. Discussion

This large cross-sectional health survey of randomly selected households, to the best of our knowledge, is the first study to date on the association between self-reported health symptoms and active UOGD sites utilizing well-defined BMPs. In adjusted models, survey respondents living within 1 mile of a multi-well UOGD site in CCOB reported greater frequencies of upper respiratory and acute response symptoms in the past 14 days than respondents living more than 2 miles from the sites. Respondents living within 2 miles of a UOGD site also reported that their children experienced greater frequencies of lower respiratory, GI and acute response symptoms in the past 14 days as well as a greater number of total symptoms, in adjusted models. We observed null results for mental health and neurological symptoms in our adjusted models. Among respondents most concerned with odors, noise and air pollution, those living within 1 mile reported greater frequencies for all types of symptoms; while among respondents least concerned with odors, noise and air pollution, those living within 1 mile reported less frequencies for all symptom types than those living more than 2 miles from the sites.

Our results are similar to previously published studies that found associations between proximity to unconventional natural gas development in Pennsylvania’s Marcellus Shale and upper respiratory symptoms [35,40,48]. However, our study is the first to report greater frequencies of self-reported nosebleeds, nausea, vomiting and shortness of breath symptoms near UOGD sites. One potential explanation is that the previous studies evaluated exposure proximity to unconventional natural gas well sites and included single well sites, while we evaluated proximity to large multi-well unconventional oil and natural gas sites in our study. Larger multi-well sites that include oil extraction may increase cumulative impacts from exposure to air pollutants, such as volatile organic compounds (VOCs), which are commonly emitted from UOGD sources and can cause a variety of acute health reactions including, but not limited to nose and throat irritation, dizziness and nausea [49].

The CCOB’s air quality monitoring system has documented numerous VOC release events from multi-well UOGD sites that were attributed to drilling and hydraulic fracturing activities during the survey period. From November–December 2021, plumes of BTEX emissions (benzene, toluene, ethylbenzene, xylenes) were frequently observed at a monitoring station within a CCOB neighborhood and were consistent with transport from UOGD sites in pre-production phases, located 1.5 miles away. On December 4, 2021, several large plumes were captured throughout the day near a UOGD site, showing elevated VOCs, including benzene levels that reached a 1-hour average estimated at 224 parts per billion (ppb) [22], exceeding the Agency for Toxic Substances and Disease Registry’s (ATSDR) federal, short-term Minimum Risk Level of 9 ppb [50]. One hour benzene averages are calculated by analyzing a one minute benzene canister sample and then applying conversion factors to each minute of the total VOC (TVOC) indicator reading for a one- hour duration [51]. This event was one of the highest total TVOC readings ever recorded in CCOB. Laboratory results of air samples confirm the plume was significantly influenced by oil and gas activity, and wind direction suggests the plume was sourced from nearby UOGD operations during the start of coiled tubing/mill out. Overall, air quality events captured by CCOB’s monitoring network lasted, on average, for 3.5 h and TVOC’s concentrations reached 23,000 ppb. Further research, which could estimate levels of pollutants for those living at various distances, could help identify potential exposure scenarios that might be linked to health outcomes.

Results from this research have important implications for future policy efforts that aim to reduce resident exposure to emissions from UOGD sites. This research calls into question the adequacy of Colorado’s current 2000 ft. setback [52] as CCOB’s air quality monitoring program has evidence of oil and gas plumes traveling over one mile and into neighborhoods. Residents in Broomfield are uniquely situated near six UOGD sites with dozens of wells, which may result in cumulative emissions exposure. Even with the most stringent BMPs in place, emissions during pre-production activities contained elevated levels of BTEX and other air toxics that may have contributed to some of the symptoms reported in our health survey. This research builds off the current body of epidemiological oil and gas literature by highlighting that cumulative emissions exposure may result from living in proximity to multiple UOGD sites. Research such as this can help inform state policymakers about BMPs and setback distances that aim to protect public health.

Interestingly, we found that a respondent’s level of concern with the top three complaints (air pollution, noise and odors) in CCOB’s oil and gas complaint database significantly modified the relationship between frequency of symptoms and setback distance. This could be due to perception bias, where those most concerned about environmental stressors are more likely to notice and/or report symptoms and those with less concerns are less likely to report symptoms or a psychosomatic effect where the anxiety and concern with pollution is causing some of these symptoms. It could also be due to recall or awareness bias in which individuals with health symptoms are more likely to remember perceived environmental exposures or notice environmental exposures. However, our mediation analysis indicates that level of environmental concerns did not differ by setback distance (see Supplemental Material, Table S2), which indicates these biases may not explain the differences in symptoms by setback distance. It also may be possible that affiliation bias (a respondent’s affiliation concerning UOGD) affected our results through either over- or under-reporting symptoms. Our cross-sectional design limits our ability to further evaluate the temporality of the bias.

Our study benefited from random selection of households with response rates evenly distributed by setback distances from CCOB’s UOGD sites and more than enough adult respondents to detect differences in frequencies of symptoms by setback distance. We were also able to adjust for many covariates that could be associated with the symptoms. We used well-established methods to evaluate the proximity of households to UOGD by using ArcGIS software 10.8.1 and geocoding the household locations for those that completed a survey.

Inherent biases in self-reporting (as previously discussed) may have affected our results. Additionally, selection bias within a household could have occurred if respondents purposely selected an individual within the household with greater (or fewer) symptoms, rather than choosing one adult (and one child, if applicable) whose birth date is closest to the date they received the survey in the mail. Participation bias may have occurred if those in favor of and/or not in favor of UOGD in CCOB or those experiencing symptoms were more or less likely to respond to the survey.

With only 59 children and fewer covariates for children, our results for children lack precision and may be biased towards the null. While we did adjust our analysis for many potential confounders, it is possible that an unexplained confounder or residual confounding is present with an unknown effect on our results. Proximity to major roadways, the COVID pandemic and source of drinking water may have affected our results. However, respondents living within 1 mile and greater than 2 miles were equally likely to be living near a major roadway (Figure 1); thus, proximity to a major roadway was not likely a major confounder. Because our survey was conducted when COVID incidence in CCOB was relatively low and COVID incidence was not associated with proximity to UOGD in CCOB, the COVID pandemic was not likely a major confounder. We also note that our stratified analysis by age and race, both known to be associated with COVID incidence, indicate that neither age or race confounded the results (see Supplemental Material, Table S6). Because CCOB lies within the Denver metro region and most residential properties are connected to municipal water sourced from outside of Broomfield [53], source of drinking was not likely a major confounder.

We did not include other populations living near the six UOGD sites in CCOB, and our results may not represent resident symptoms outside of CCOB. Some residents in adjacent Adams County were among the closest individuals living to pre-production development. While including counties with residents living in close proximity to CCOB’s UOGD would have improved precision, it is not possible to know how it would affect our results. Including all residents within proximity to UOGD regardless of jurisdictional boundaries would improve the representativeness and generalizability of future studies.

## 5. Conclusions

Our results indicate that people living within 1 mile of multi-well unconventional oil and natural gas sites more frequently report upper respiratory and acute (e.g., nosebleeds, nausea, shortness of breath) symptoms than people living more than 2 miles from the sites. Because our cross-sectional design does not provide temporal information, it is not possible to determine if proximity to UOGD caused any of the reported symptoms. A possible explanation for the increase in symptoms reported near oil and gas sites could include cumulative and additive impacts from exposure to emissions from multiple UOGD sites. However, other explanations are possible. Additional study using more precise estimates of exposure and objective measures of health outcomes, as well as qualitative designs with focus groups would be useful to better understand the relationship between proximity to UOGD and the health symptoms reported in this study.

## Figures and Tables

**Table 1:** Demographics of adult survey respondents and household distance from well site.

Parameter	Within 1 Mile	1–2 Miles	>2 Miles	Total
Number of survey respondents (response rate [%])	61 (11.6)	69 (10.0)	297 (10.7)	427 (10.7)
	Age in Years (%)			
18–44	18	5.9	23.9	21.8
45–54	8.2	29	20.2	19.9
55–64	14.8	29	20.5	21.1
65–74	41	14.5	26.9	26.9
≥75	18	11.6	8.5	10.3
White alone (%) ^1^	89.9	90.2	90.9	90.6
Female (%)	44.3	44.9	59.3	54.8
Never smoked or lived with someone who smoked (%)	88.5	89.9	82.5	84.5
Average Days of Exercise per Week	3.9	4.7	3.5	3.8
	Average number of alcoholic drinks per week (%)			
None	31.1	44.9	40.7	40.1
1–2	27.9	17.4	23.6	23.2
3–5	19.7	20.3	9.5	19.7
6–10	13.1	14.5	11.1	11.9
>10	8.2	2.9	5.1	5.2
	Average Hours Spent in Home Per Day (%)			
Less than 8	6.6	5.8	1.4	2.8
8–12	19.7	14.5	12.1	13.6
13–15	16.4	24.6	20.9	20.8
16–20	47.5	21.7	31	31.9
≥21	9.8	33.3	34.7	30.9
<2 years in household	19.7	5.8	7.7	9.1
	Occupation (%)			
Management, professional and related occupations	29.5	42.0	52.5	47.5
Service, sales, office, natural resources, construction, maintenance, production, transportation and material moving occupations ^2^	11.5	8.7	9.1	9.4
Not working (retired, homemaker, student, unemployed)	59.0	49.3	38.4	43.1
Mean Likert Score for Concerns about air, noise and water	4.9	4.8	4.6	4.7

^1^ Percentage of respondents that identified as white. Approximately 10% of respondents identified as either Asian alone (2.6%), Hispanic/Latino alone (2.1%), American Indian/Alaska Native, Native Hawaiian/other Pacific Islander, Asian/White, Hispanic/Latino/White, Black/White or other. No survey respondents identified as Black or African American alone. ^2^ No respondents reported that they worked in the oil and gas industry.

**Table 2:** Unadjusted model for difference in means for survey respondents living more than 2 miles, 1–2 miles and <1 mile from a multi-well oil and gas site in Broomfield Colorado, October–December 2021.

Outcome	Unadjusted Model Main Analysis (N = 427)	
	Difference between >2 Mile and 1–2 Mile Means (LCL, UCL)	Difference between >2 Mile and <1 Mile Means (LCL, UCL)
Total Number of Symptoms (N)	−0.31 (−1.57, 0.96)	−0.44 (−1.77, 0.89)
Total summed Likert Score	−0.92 (−3.14, 1.67)	−1.01 (−3.34, 1.71)
^1^ Upper Respiratory (summed Likert Score)	−0.40 (−1.06, 0.41)	0.15 (−0.62, 1.09)
^2^ Lower Respiratory (summed Likert Score)	0.03 (−0.16, 0.25)	0.04 (−0.16, 0.28)
^3^ Mental Health (summed Likert Score)	−0.06 (−0.82, 0.88)	−0.88 (1.55, 0.04)
^4^ Neurological (summed Likert Score)	−0.34 (−1.10, 0.57)	−0.39, (−1.18, 0.56)
^5^ Gastrointestinal (summed Likert Score)	−0.003 (−0.13, 0.14)	0.020 (−0.12, 0.18)
^6^ Acute (summed Likert Score)	−0.13 (−0.70, 0.53)	0.17 (−0.46, 0.90)

^1^ Upper Respiratory = cough + nasal congestion + runny nose + throat irritation + nosebleeds. ^2^ Lower Respiratory = short breath + lung irritation. ^3^ Mental Health = anxiety stress + diff sleeping + difficulty concentrating + lack energy fatigue. ^4^ Neurological = dizziness + difficulty concentrating + headaches + numbness tingling + ringing ears hearing loss + muscle aches weakness pain. ^5^ Gastrointestinal = nausea + vomiting. ^6^ Acute = nausea + vomiting + Nosebleeds + lung irritation + short breath +cough + throat irritation. LCL = lower 95% confidence level, UCL = upper 95% confidence level.

**Table 3:** Difference in means for survey respondents living more than 2 miles, 1–2 miles, and <1 mile from a multi-well oil and gas site in Broomfield Colorado, October–December 2021 ^1^.

Outcome	Main Analysis (N = 427)		Sum Likert Score for Odors, Noise and Air in Top 50th Percentile (≥4, N = 239)		Sum Likert Score for Odors, Noise and Air in below the 50th Percentile (<4, N = 188)	
	Difference between >2 Mile and 1–2 Mile Means (LCL, UCL)	Difference between >2 Mile and <1 Mile Means (LCL, UCL)	Difference between >2 Mile and 1–2 Mile Means (LCL, UCL)	Difference between >2 Mile and <1 Mile Means (LCL, UCL)	Difference between >2 Mile and 1–2 mile Means (LCL, UCL)	Difference between >2 Mile and <1 Mile Means (LCL, UCL)
Total Number of Symptoms (N)	0.31 (−0.94, 1.55)	0.70 (−0.62, 2.02)	−0.05 (−1.59, 1.49)	***2.88*** ***(1.14, 4.63)***	0.25 (−1.60, 2.05)	−1.51 (−3.26, 0.23)
Total summed Likert Score	0.56 (−1.64, 4.53)	1.57 (−1.04, 6.20)	−0.37 (−2.92, 5.32)	***8.53*** ***(2.39, 20.17)***	0.61 (−1.99, 6.44)	−3.09 (−4.23, 0.33)
^2^ Upper Respiratory (summed Likert Score)	−0.15 (−0.63, 0.97)	0.81 (0.06, 2.58)	−0.34 (−0.93, 1.64)	***3.16*** ***(0.82, 8.60)***	−0.18 (−0.66, 1.34)	−0.58 (−0.87, 0.54)
^3^ Lower Respiratory (summed Likert Score)	0.09 (−0.11 0.47)	0.18 (−0.06, 0.63)	0.15 (−0.14, 0.87)	***0.51*** ***(0.03, 1.62)***	−0.11 (−0.27, 0.26)	−0.13 (−0.28 0.22)
^4^ Mental Health (summed Likert Score)	0.88 (−0.31, 3.43)	0.24 (−0.75, 2.53)	0.18 (−0.96, 3.45)	***2.62*** ***(0.21, 8.45)***	1.21 (−0.40, 6.10)	−1.33 (−2.44, 0.42)
^5^ Neurological (summed Likert Score)	0.13 (−0.50, 1.42)	0.05 (−0.59, 1.38)	−0.03 (−0.74, 1.86)	***1.42*** ***(0.001, 4.68)***	0.07 (−0.72, 2.27)	−1.01 (−−1.29, 0.25))
^6^ Gastrointestinal (summed Likert Score)	0.03 (−0.09, 0.23)	0.09 (−0.05, 0.32)	0.01 (−0.17, 0.26)	***0.30*** ***(0.01, 0.84)***	−0.03 (−0.14, 0.19)	−0.005 (−0.12, 0.21)
^7^ Acute (summed Likert Score)	0.09 (−0.44, 1.03)	***0.75*** ***(0.004, 1.99)***	−0.027 (−0.79, 1.64)	***2.76*** ***(0.89, 6.24)***	−0.07 (−0.55, 0.94)	−0.47 (−0.81, 0.30)

^1^ Adjusted for age, sex, race, smoking, alcohol consumption, time spent in home, number of children <18 years in home, exercise, number of chronic health conditions, time of residence at current home, education level and occupation. ^2^ Upper Respiratory = cough + nasal congestion + runny nose + throat irritation + nosebleeds. ^3^ Lower Respiratory = short breath + lung irritation. ^4^ Mental Health = anxiety stress + diff sleeping + difficulty concentrating + lack energy fatigue. ^5^ Neurological = dizziness + difficulty concentrating + headaches + numbness tingling + ringing ears hearing loss + muscle aches weakness pain. ^6^ Gastrointestinal = nausea + vomiting. ^7^ Acute = nausea + vomiting + nosebleeds + lung irritation + short breath +cough + throat irritation. LCL = lower 95% confidence level, UCL = upper 95% confidence level. **Bold Italics** indicate statistically significant results at a *p*-value < 0.05.

**Table 4:** Difference in means for 59 children living more than 2 miles and <1 mile from a multi-well oil and gas site in Broomfield Colorado, October–December 2021 ^1^.

Outcome	Difference between >2 Mile and <1 Mile Means (LCL, UCL)
Total Number of Symptoms (N)	***2.29 (0.05, 4.53)***
Total summed Likert Score	3.99 (−1.65, 9.64)
^2^ Upper Respiratory (summed Likert Score)	1.63 (−0.63, 3.89)
^3^ Lower Respiratory (summed Likert Score)	***0.83 (0.12, 1.54)***
^4^ Mental Health (summed Likert Score)	0.06 (−1.81, 1.93)
^5^ Neurological (summed Likert Score)	−0.36 (−1.87, 1.14)
^6^ Gastrointestinal (summed Likert Score)	***0.81 (0.3, 1.31)***
^7^ Acute (summed Likert Score)	***2.38 (0.36, 4.41)***

^1^ Adjusted for age, sex, smoking, number of children <18 years in home, number of chronic conditions. ^2^ Upper Respiratory = cough + nasal congestion + runny nose + throat irritation + nosebleeds. ^3^ Lower Respiratory = short breath + lung irritation. ^4^ Mental Health = anxiety stress + diff sleeping + difficulty concentrating + lack energy fatigue. ^5^ Neurological = dizziness + difficulty concentrating + headaches + numbness tingling + ringing ears hearing loss + muscle aches weakness pain. ^6^ Gastrointestinal = nausea + vomiting. ^7^ Acute = nausea + vomiting + nosebleeds + lung irritation + short breath + cough + throat irritation. LCL = lower 95% confidence level, UCL = upper 95% confidence level. **Bold Italics** indicate statistically significant results at a *p*-value < 0.05.

## Data Availability

The data presented in this study are openly available at https://www.broomfieldvoice.com/oil-and-gas-health-survey (accessed on 1 July 2022).

## Supplementary Materials

The following supporting information can be downloaded at: https://www.mdpi.com/article/10.3390/ijerph20032634/s1, Figure S1: Survey; Figure S2: Postcard; Table S1: Frequency of health symptoms (unadjusted) for adults within the past 14 days and distance from unconventional oil and gas development; Table S2: Mediation analysis for adult survey respondents living more than 2 miles, 1–2 miles, and <1 mile from a multi-well oil and gas site in Broomfield Colorado, October–December 2021: Association between distance band and summed Likert score for oil and gas environmental concerns (air pollution, noise and odors); Table S3: Mediation analysis for adult survey respondents living more than 2 miles, 1–2 miles and <1 mile from a multi-well oil and gas site in Broomfield Colorado, October–December 2021: Association between Likert scores for reported health symptoms and summed Likert score for oil and gas environmental concerns (air pollution, noise and odors); Table S4: Difference in means for adult survey respondents living more than 2 miles, 1–2 miles and <1 mile from a multi-well oil and gas site in Broomfield Colorado, October–December 2021: Sensitivity Analysis for respondents at home more than 12 h a day and stratified analysis Likert score for environmental concerns other than oil and gas in the upper and lower 50th percentile; Table S5: Difference in means for adult survey respondents living more than 2 miles, 1–2 miles and <1 mile from a multi-well oil and gas site in Broomfield Colorado, October–December 2021: Stratified by gender and chronic conditions; Table S6: Difference in means for adult survey respondents living more than 2 miles, 1–2 miles and <1 mile from a multi-well oil and gas site in Broomfield Colorado, October–December 2021: Sensitivity Analysis for respondents identifying as white, never smoked or lived with a smoker, 55 years and older and lived in their home for 2 or more years.
