# Supplementary material for: Health Symptoms and Proximity to Active Multi-Well Unconventional Oil and Gas Development Sites in the City and County of Broomfield, Colorado
Source: Int J Environ Res Public Health. 2023 Feb 1;20(3):2634. doi: 10.3390/ijerph20032634 (PMC9915243; doi:10.3390/ijerph20032634)
Supplement: Supplementary file 1 [file ijerph-20-02634-s001.zip › Post Card.pdf]

YOUR HEALTH

SURVEY IN BROOMFIELD

Your household has been randomly selected to participate in a survey understanding the health of residents living in the City and County of Broomfield. This research is funded by the City and County of Broomfield Department of Public Health & Environment and is being conducted in collaboration with the Colorado School of Public Health at University of Colorado Denver Anschutz campus.

This survey will take about 20-30 minutes to complete. The questions will ask for health and demographic information to better understand resident health near and away from oil and gas activity. After completing this survey, you can choose to enter into a drawing for a chance to win a $50 Visa gift card. Only one adult per household can complete the survey. Please select the adult whose birthday is closest to the date you received this survey in the mail.

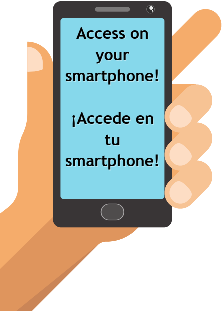

Su hogar ha sido seleccionado al azar para participar en una encuesta sobre la salud de los residentes que viven en la ciudad y el condado de Broomfield. Esta investigación está financiada por el Departamento de Salud Pública y Medio Ambiente de la Ciudad y el Condado de Broomfield y se está llevando a cabo en colaboración con la Escuela de Salud Pública de Colorado en el campus Anschutz de la Universidad de Colorado en Denver.

Esta encuesta tardará entre 20 y 30 minutos en completarse. Las preguntas solicitarán información demográfica y de salud para comprender mejor la salud de los residentes cerca y lejos de la actividad de petróleo y gas. Después de completar esta encuesta, puede optar por participar en un sorteo para tener la oportunidad de ganar una tarjeta de regalo Visa de $ 50. Solo un adulto por hogar puede completar la encuesta. Seleccione el adulto cuyo cumpleaños sea más cercano a la fecha en que recibió esta encuesta por correo.

City and County of Broomfield
One DesCombes Drive
Broomfield, CO 80020
Broomfield.org

PLACEHOLDER FOR POSTAGE

PLACEHOLDER FOR QR CODE

This QR code is unique to your household. To scan the code, open the camera app on your smartphone, point the camera at the code, and then tap the pop-up notification. If you have questions or cannot access the survey through the QR code, contact Broomfield Department of Public Health and Environment at 720.887.2200 or send an email to publichealth@broomfield.org.

Este código QR es exclusivo de su hogar. Para escanear el código, abra la aplicación de la cámara en su teléfono inteligente, apunte la cámara al código y luego toque la notificación emergente. Si tiene preguntas o no puede acceder a la encuesta a través del código QR, comuníquese con el Departamento de Salud Pública y Medio Ambiente de Broomfield al 720.887.2200 o envíe un correo electrónico a publichealth@broomfield.org.

DEADLINE/PLAZO: XXX
