# Supplementary material for: Health Symptoms and Proximity to Active Multi-Well Unconventional Oil and Gas Development Sites in the City and County of Broomfield, Colorado
Source: Int J Environ Res Public Health. 2023 Feb 1;20(3):2634. doi: 10.3390/ijerph20032634 (PMC9915243; doi:10.3390/ijerph20032634)
Supplement: Supplementary file 1 [file ijerph-20-02634-s001.zip › Survey.pdf]

City and County of Broomfield Health Symptoms Survey

This survey will focus on understanding the health of residents living across the City and County of Broomfield. This research is funded by the City and County of Broomfield Public Health Department and is being conducted in collaboration with University of Colorado School of Public Health. The goal of this research is to understand resident health in communities near and away from oil and gas operations.

We ask that only one adult completes the survey per household. Please select the adult whose birthday is closest to the date you received this survey in the mail. The participating adult must live full time in the household.

1. Survey Consent

1. Title of Research: City and County of Broomfield Health Symptoms Survey
2. Investigators: This research is being conducted under the direction of Meagan L. Weisner, PhD, City and County of Broomfield, Senior Environmental Epidemiologist, with assistance from Lisa McKenzie, PhD, MPH, University of Colorado School of Public Health and William Allshouse, PhD, University of Colorado School of Public Health.
3. Purpose: This survey will focus on enhancing data collection by gathering a range of health symptoms from residents living across the City and County of Broomfield (CCOB). This survey is designed to help CCOB understand resident health in communities near and away from oil and gas operations.
4. Procedures: This survey will take about 20 minutes to complete. The questions will ask about recent health symptoms you may have experienced and demographic questions. At any time, you may choose to discontinue the survey if you wish to do so. This survey is voluntary and there is no compensation for your time. However, you may choose to be entered into a drawing for a chance to win a $50 Visa gift card.
5. Risks: This survey presents minimal risks to you, but no more than one would expect in everyday life. Potential risks include a breach in data confidentiality.
6. Benefits: You may not directly benefit from this study, but the findings will contribute to research that aims to understand health symptoms across CCOB and proximity to oil
7. Data Collection & Storage: Any information collected about you will be kept secure, and only the people working with the study will see the data, unless required by law. This survey will be administered using Survey123. The data will be securely stored on HIPAA compliant servers at the City and County of Broomfield and is protected by a password. All electronic surveys will be destroyed after seven years. We may publish what we learn from this study but we will not reveal your household address or your identity. The data we collect will be used for this study, but may also be important for future research. Your data may be used for future research or distributed to other researchers for future study without additional consent if information that identifies you is removed from the data.
8. Contact Information: If you have questions about the study, you should call or email the principal investigator, Meagan L. Weisner, PhD at mweisner@broomfield.org , phone: 720-887-2292. You may also contact Lisa McKenzie, PhD, MPH at lisa.mckenzie@cuanschutz.edu, phone 303-724-5557 or the Colorado Multi-Institutional Review Board at (the responsible Institutional Review Board) at (303) 724-1055.
9. Consent Statement: I have read the above information describing this study. I am 18 years of age or older and freely consent to participate. I understand that I am free to withdraw from this study at any time without penalty. By continuing to complete the survey, I am giving consent.

2. What is your physical address?*

Enter the street number, street name, unit number (if applicable), city, state, and zip code where you live. Once you see a marker appear on the correct location, move on to the next question.

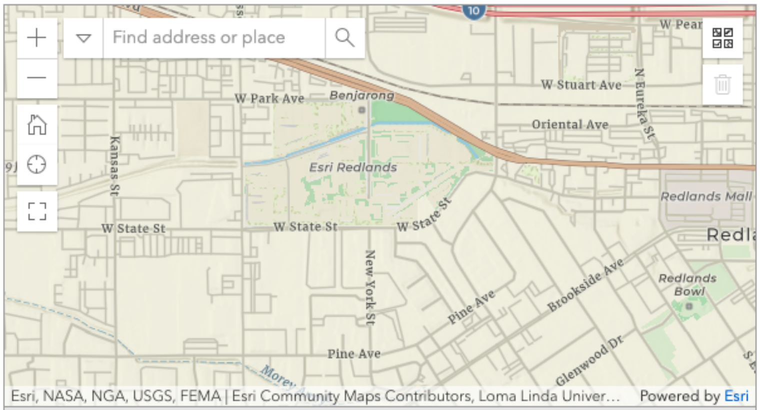

No geometry captured yet.

3. How long have you lived at your current location?*

-Please Select-

- less than 2 years
- 2 - 5 years
- 5 - 20 years
- greater than 20 years

r household (includin

4. How many individuals live in your household (including yourself)?*

-Please Select-

- 1
- 2
- 3
- 4

usehold are under the age of 18?*

ern to the City and County of

4. How many individuals live in your household (including yourself)?*

-Please Select-

- 5
- 6
- 8
- 9

usehold are under the age of 18?*

4. How many individuals live in your household (including yourself)?*

-Please Select-

8

9

10

greater than 10

usehold are under the age of 18?*

ern to the City and County of

5. How many individuals in your household are under the age of 18?*

-Please Select-

0

1

2

3

ern to the City and County of

5. How many individuals in your household are under the age of 18?*

-Please Select-

4

5

6

8

ern to the City and County of

5. How many individuals in your household are under the age of 18?*

-Please Select-

8

9

10

greater than 10

ern to the City and County of

6. Have you ever filed a health concern to the City and County of Broomfield?*

- ◯ Yes
- ◯ No
- ◯ Unsure

7. In the past 14 days, indicate how often you have experienced the following symptoms?

Please check appropriate box for each symptom.

	Never	Once within 14 days	Two to five times within 14 days	Five to thirteen times within 14 days	Every day
**Difficulty Sleeping***	◯	◯	◯	◯	◯
**Headaches***	◯	◯	◯	◯	◯
**Dizziness***	◯	◯	◯	◯	◯
**Anxiety or Stress***	◯	◯	◯	◯	◯
**Numbness/tingling***	◯	◯	◯	◯	◯
**Nausea***	◯	◯	◯	◯	◯
**Vomiting***	◯	◯	◯	◯	◯
**Nosebleeds***	◯	◯	◯	◯	◯

Ringing in ears or hearing loss*	○	○	○	○	○
Shortness of breath*	○	○	○	○	○
Cough*	○	○	○	○	○
Runny nose*	○	○	○	○	○
Nasal congestion*	○	○	○	○	○
Lung irritation*	○	○	○	○	○
Throat irritation*	○	○	○	○	○
Eye irritation*	○	○	○	○	○
Skin irritation*	○	○	○	○	○
Muscle aches, weakness, or pain*	○	○	○	○	○
Difficulty concentrating*	○	○	○	○	○
Lack of energy/fatigue*	○	○	○	○	○

8. In the past 14 days, did you seek medical attention for any of the symptoms you experienced?*

- ○ Yes
- ○ No
- ○ Unsure
- ○ I did not experience any symptoms

9. In the past 14 days, did any of your symptoms interfere with your daily routine (for example, missed work or school, couldn't perform daily tasks)?*

- ○ Yes
- ○ No
- ○ Unsure
- ○ I did not experience any symptoms

10. In the past two years, have you experienced any of the following symptoms?*

Check all that apply.

- ☐ Difficulty sleeping
- ☐ Headache
- ☐ Dizziness
- ☐ Anxiety or Stress
- ☐ Numbness/tingling
- ☐ Nausea
- ☐ Vomiting
- ☐ Nosebleed
- ☐ Ringing in ears or hearing loss
- ☐ Shortness of breath
- ☐ Cough
- ☐ Runny nose
- ☐ Nasal congestion
- ☐ Lung irritation
- ☐ Throat irritation
- ☐ Eye irritation
- ☐ Skin irritation
- ☐ Skin irritation
- ☐ Muscle aches, weakness, or pain
- ☐ Difficulty concentrating
- ☐ Lack of energy/fatigue
- ☐ I have not experienced any of these symptoms

11. In the past five years, have you experienced any of the following symptoms?*

Check all that apply.

- [ ] Difficulty sleeping
- [ ] Headache
- [ ] Dizziness
- [ ] Anxiety or Stress
- [ ] Numbness/tingling
- [ ] Nausea
- [ ] Vomiting
- [ ] Nosebleed
- [ ] Ringing in ears or hearing loss
- [ ] Shortness of breath
- [ ] Cough
- [ ] Runny nose
- [ ] Nasal congestion
- [ ] Lung irritation
- [ ] Throat irritation
- [ ] Eye irritation
- [ ] Skin irritation
- [ ] Muscle aches, weakness, or pain
- [ ] Difficulty concentrating
- [ ] Lack of energy/fatigue
- [ ] I have not experienced any of these symptoms

12. Please check any of the following health conditions that you currently have:*

Check all that apply.

- ☐ Asthma
- ☐ Autoimmune disease
- ☐ Cancer
- ☐ COPD
- ☐ High Blood Pressure
- ☐ Thyroid disease
- ☐ Type I diabetes
- ☐ Type II diabetes
- ☐ I do not have any health conditions
- ☐ Other

13. Are you pregnant?*

- ○ Yes
- ○ No
- ○ Not applicable

14. Have you had a trip to the Emergency Room in the past 14 days?*

- ○ Yes
- ○ No

15. If yes, what was your trip to the Emergency Room for?

Skip this question if the answer to question 14 is no.

16. Do you want to fill this out for a child in your household?*

If you have more than one child, please only fill this out for the child whose birthday is closest to the date you received this survey in the mail. Please do not fill this out for a child under two years of age. The child must reside primarily in your home and does not reside in another location on most days. You must be the parent or legal guardian of the child to complete the following questions.

- ○ Yes
- ○ No
- ○ Not applicable

17. What is the age of your child?

- ○ 2-5 years
- ○ 6-10 years
- ○ 11-14 years
- ○ 15-18 years

18. What gender does your child identify with?

- ○ Male
- ○ Female
- ○ My child identifies in another way

19. Please check any of the following health conditions that your child currently has:

- ☐ Asthma
- ☐ Autoimmune disease
- ☐ Cancer
- ☐ Thyroid disease
- ☐ Type I diabetes
- ☐ Type II diabetes
- ☐ My child does not have any health conditions
- ☐ Other

20. In the past 14 days, indicate how often your child has experienced the following symptoms?

Please check appropriate box for each symptom.

	Never	Once within 14 days	Two to five times within 14 days	Five to thirteen times within 14 days	Every day
**Difficulty Sleeping**	○	○	○	○	○
**Headaches**	○	○	○	○	○
**Dizziness**	○	○	○	○	○
**Anxiety or Stress**	○	○	○	○	○
**Numbness/tingling**	○	○	○	○	○
**Nausea**	○	○	○	○	○
**Vomiting**	○	○	○	○	○
**Nosebleeds**	○	○	○	○	○

Ringing in ears or hearing loss	○	○	○	○	○
Shortness of breath	○	○	○	○	○
Cough	○	○	○	○	○
Runny nose	○	○	○	○	○
Nasal congestion	○	○	○	○	○
Lung irritation	○	○	○	○	○
Throat irritation	○	○	○	○	○
Eye irritation	○	○	○	○	○
Skin irritation	○	○	○	○	○
Muscle aches, weakness, or pain	○	○	○	○	○
Difficulty concentrating	○	○	○	○	○

21. What is your age?*

- ○ 18-25
- ○ 26-34
- ○ 35-44
- ○ 45-54
- ○ 55-64
- ○ 65-74
- ○ 75-84
- ○ Greater than 85 years

22. What gender do you identify with?

- Male
- Female
- I identify in another way

23. Do you use tobacco or marijuana products regularly?*

- Yes
- No
- Used to, but no longer do
- Prefer not to answer

24. Does anyone in your household smoke tobacco or marijuana regularly inside the home?*

- Yes
- No
- Used to, but no longer does
- Prefer not to answer

25. On average, how many days per week do you exercise (30 or more minutes)?*

- None
- 1
- 2
- 3
- 4
- 5
- 6
- 7

26. On average, how many alcoholic drinks do you consume each week?*

- ○ None
- ○ 1-2
- ○ 3-5
- ○ 6-10
- ○ more than 10

27. What is your occupation?*

For example: homemaker, high school teacher, sales associate, registered nurse, etc.

28. On average, how many hours per day are you in your home (including sleeping)?*

-Please Select-

- Less than 8
- 8-12 hours
- 13-15 hours
- 16-20 hours

ucation?*

age

28. On average, how many hours per day are you in your home (including sleeping)?*

-Please Select-

- 8-12 hours
- 13-15 hours
- 16-20 hours
- 21 or more hours

ucation?*

ege

29. What is your highest level of education?*

- ○ Less than high school diploma
- ○ High school diploma/GED, some college
- ○ Vocational degree, Bachelor's degree
- ○ Advanced degree (for example, Master's degree, PhD, MD, JD)

30. What racial or ethnic group(s) do you most identify with?*

Select all that apply.

- ☐ Black or African-American
- ☐ American Indian or Alaska Native
- ☐ Asian
- ☐ Native Hawaiian or other Pacific Islander
- ☐ Hispanic/Latino
- ☐ White/Caucasian
- ☐ Other

31. Please indicate how concerned you are with the following environmental issues near your place of residence:

	Not at all	Slightly	Somewhat	Moderately	Extremely
**Water***	○	○	○	○	○
**Air***	○	○	○	○	○
**Odor***	○	○	○	○	○
**Noise***	○	○	○	○	○
**Lighting***	○	○	○	○	○
**Dust***	○	○	○	○	○
**Traffic***	○	○	○	○	○
**Oil Spill***	○	○	○	○	○
**Wildlife***	○	○	○	○	○
**Waste***	○	○	○	○	○

32. Would you be interested in participating in a focus group?*

You will not be contacted for anything other than follow up research related to this survey.

○ Yes

○ No
