# Supplementary material for: Health Symptoms and Proximity to Active Multi-Well Unconventional Oil and Gas Development Sites in the City and County of Broomfield, Colorado
Source: Int J Environ Res Public Health. 2023 Feb 1;20(3):2634. doi: 10.3390/ijerph20032634 (PMC9915243; doi:10.3390/ijerph20032634)
Supplement: Supplementary file 1 [file ijerph-20-02634-s001.zip › Tables S1-S6.pdf]

Table S1. Frequency of health symptoms (unadjusted) for adults within the past 14 days and distance from unconventional oil and gas development.

Sum Likert Scores [mean ± SD]	Within 1 Mile	1-2 Miles	Greater than 2 Miles	Total Population
Total number of symptoms reported at least once in last 14-days (N)	6.1 ± 5.3	6.3 ± 5.2	6.6 ± 4.6	6.5 ± 4.8
All Symptoms	11.7 ± 11.8	11.7 ± 11.6	12.4 ± 10.5	12.2 ± 10.9
Upper Respiratory	3.5 ± 3.8	2.9 ± 3.8	3.2 ± 3.6	3.2 ± 3.6
- Cough	0.7 ± 1.1	0.5 ± 0.9	0.6 ± 1	0.6 ± 1
- Nasal Congestion	1.1 ± 1.2	0.9 ± 1.2	1 ± 1.2	1 ± 1.2
- Runny Nose	1 ± 1.3	0.9 ± 1.1	1 ± 1.2	1 ± 1.2
- Throat Irritation	0.4 ± 0.9	0.4 ± 0.8	0.4 ± 0.8	0.4 ± 0.8
- Nosebleeds	0.3 ± 0.6	0.2 ± 0.7	0.2 ± 0.6	0.2 ± 0.6
Lower Respiratory	0.5 ± 1.2	0.5 ± 1.1	0.5 ± 1.3	0.5 ± 1.2
- Shortness of Breath	0.4 ± 0.8	0.3 ± 0.8	0.3 ± 0.7	0.3 ± 0.7
- Lung Irritation	0.2 ± 0.5	0.2 ± 0.5	0.2 ± 0.7	0.2 ± 0.7
Mental	3.2 ± 3.5	4.1 ± 3.8	4.1 ± 3.7	4 ± 3.7
- Anxiety or Stress	0.8 ± 1.1	1.2 ± 1.2	1.2 ± 1.2	1.1 ± 1.2
- Difficulty Sleeping	1.3 ± 1.3	1.3 ± 1.1	1.3 ± 1.2	1.3 ± 1.2
- Difficulty Concentrating	0.4 ± 0.8	0.6 ± 1	0.6 ± 1.1	0.6 ± 1
- Lack of Energy or Fatigue	0.7 ± 1.1	1 ± 1.1	1 ± 1.2	0.9 ± 1.2
Neurological	3.5 ± 3.9	3.6 ± 4.2	3.9 ± 3.9	3.8 ± 3.9
- Dizziness	0.2 ± 0.6	0.4 ± 0.8	0.3 ± 0.8	0.3 ± 0.7
- Headaches	0.8 ± 1	0.7 ± 0.8	0.8 ± 0.9	0.8 ± 0.9
- Numbness or Tingling	0.4 ± 0.9	0.4 ± 0.9	0.4 ± 0.9	0.4 ± 0.9

- Ringing in Ears or Hearing Loss	0.9 ± 1.5	0.8 ± 1.4	1 ± 1.5	1 ± 1.5
- Muscle Aches, Weakness or Pain	0.8 ± 1.2	0.7 ± 1.2	0.8 ± 1.2	0.8 ± 1.2
Gastrointestinal	0.3 ± 0.8	0.3 ± 0.7	0.3 ± 0.7	0.3 ± 0.7
- Nausea	0.3 ± 0.7	0.2 ± 0.6	0.3 ± 0.6	0.3 ± 0.6
- Vomiting	0.1 ± 0.2	0.1 ± 0.2	0.044 ± 0.2	0.05 ± 0.2
Irritation				
- Skin Irritation	0.4 ± 1	0.4 ± 0.9	0.3 ± 0.7	0.3 ± 0.8
- Eye Irritation	0.8 ± 1	0.6 ± 1	0.7 ± 1.1	0.7 ± 1
Acute	2.3 ± 3.3	1.9 ± 3	2 ± 3	2 ± 3.1

We evaluated for mediation by using multiple linear regression with (1) the mediator (sum of Likert scores for air pollution, noise, and odor concerns) as the dependent variable and setback distance as the main predictor (Table S2); (2) the sum of Likert scores for a group of health symptoms as the dependent variable and the mediator as the main predictor (Table S3); and (3) the sum of Likert scores for a group of health symptoms as the dependent variable and setback distance as the main predictor (Main Text Table 3) [47]. We did not observe a statistically significant association between distance bands and the sum of Likert scores for air pollution, noise, and odor concerns. Because all three regressions did not return statistically significant results for the main predictor, mediation is not indicated [48].

Table S2. Mediation analysis for adult survey respondents living more than 2 miles, 1-2 miles, and < 1 mile from a multi-well oil and gas site in Broomfield Colorado, October -December 2021: Association between distance band and summed Likert score for oil and gas environmental concerns (air pollution, noise and odors).

Outcome (Dependent variable)	Difference between > 2 mile and 1-2 mile means (LCL, UCL)	Difference between > 2 mile and < 1 mile means (LCL, UCL)
Summed Likert score for oil and gas environmental concerns[a]	0.48 (-0.58, 1.53)	0.35 (-0.64, 1.35)

[a]Adjusted for age, sex, race, smoking, alcohol consumption, time spent in home, number of children <18 years in home, exercise, number of chronic health conditions, time of residence at current home, education level, and occupation.

LCL = lower 95% confidence level, UCL = upper 95% confidence level.

We evaluated for mediation by using multiple linear regression with (1) the mediator (sum of Likert scores for air pollution, noise, and odor concerns) as the dependent variable and setback distance as the main predictor (Table S2); (2) the sum of Likert scores for a group of health symptoms as the dependent variable and the mediator as the main predictor (Table S3); and (3) the sum of Likert scores for a group of health symptoms as the dependent variable and setback distance as the main predictor (Main Text Table 3) [47]. We did not observe a statistically significant association between distance bands and the sum of Likert scores for air pollution, noise, and odor concerns. Because all three regressions did not return statistically significant results for the main predictor, mediation is not indicated [48].

Table S3. Mediation analysis for adult survey respondents living more than 2 miles, 1-2 miles, and < 1 mile from a multi-well oil and gas site in Broomfield Colorado, October -December 2021: Association between Likert scores for reported health symptoms and summed Likert score for oil and gas environmental concerns (air pollution, noise and odors).

Main Predictor	Total Number of Symptoms (N) (UCL, LCL)	Total summed Likert Score (UCL, LCL)	[2]Upper Respiratory (summed Likert Score) (UCL, LCL)	[3]Lower Respiratory (summed Likert Score) (UCL, LCL)	[4]Mental Health (summed Likert Score) (UCL, LCL)	[5]Neurological (summed Likert Score) (UCL, LCL)	[6]Gastrointestinal (summed Likert Score) (UCL, LCL)	[7]Acute (summed Likert Score) (UCL, LCL)
Summed Likert score for oil and gas environmental concerns[a]	0.50 (0.39, 0.61)	0.98 (0.60, 1.54)	0.22 (0.11, 0.41)	0.06 (0.03, 0.11)	0.41 (0.21, 0.73)	0.24 (0.13, .38)	0.01 (-0.002, 0.032)	0.19 (0.11, 0.31)

[a]Adjusted for age, sex, race, smoking, alcohol consumption, time spent in home, number of children <18 years in home, exercise, number of chronic health conditions, time of residence at current home, education level, and occupation. [2]Upper Respiratory = cough + nasal_congestion+ runny_nose + throat_irritation +nosebleeds. [3]Lower Respiratory = short_breath + lung_irritation. [4]Mental Health = anxiety_stress+ diff_sleeping + difficulty_concentrating + lack_energy_fatigue. [5]Neurological = dizziness + difficulty_concentrating + headaches + numbness_tingling + ringing_ears__hearing_loss + muscle_aches_weakness_pain. [6]Gastrointestinal = nausea + vomiting. [7]Acute = nausea + vomiting + Nosebleeds + lung_irritation + short_breath +cough + throat_irritation.

LCL = lower 95% confidence level, UCL = upper 95% confidence level.

Table S4. Difference in means for adult survey respondents living more than 2 miles, 1-2 miles, and < 1 mile from a multi-well oil and gas site in Broomfield Colorado, October -December 2021: Sensitivity Analysis for respondents at home more than 12 hours a day and stratified analysis Likert score for environmental concerns other than oil and gas in the upper and lower 50th percentile[a]

	At home for more than 12 hours per day (N = 268)		Likert score for environmental concerns other than oil and gas in the upper 50th percentile (N = 221)		Likert score for environmental concerns other than oil and gas in the lower 50th percentile (N= 206)	
Outcome	Difference between > 2 mile and 1-2 mile means (LCL, UCL)	Difference between > 2 mile and < 1 mile means (LCL, UCL)	Difference between > 2 mile and 1-2 mile means (LCL, UCL)	Difference between > 2 mile and < 1 mile means (LCL, UCL)	Difference between > 2 mile and 1-2 mile means (LCL, UCL)	Difference between > 2 mile and < 1 mile means (LCL, UCL)
Total Number of Symptoms (N)	0.61 (-1.05, 2.27)	0.99 (-0.76, 2.73)	-0.27 (-2.06, 1.53)	1.72 (-0.14, 3.57)	0.85 (-0.64, 2.33)	-1.09 (-2.77, 0.58)
Total summed Likert Score	1.15 (-1.72, 8.03)	2.30 (-1.16, 10.31)	-1.19 (-3.67, 5.23)	4.96 (-0.06, 15.68)	1.87 (-1.00, 7.42)	-2.39 (-4.09, 1.60)
[2]Upper Respiratory (summed Likert Score)	-0.36 (-0.82, 1.60)	0.89 (-0.25, 4.30)	-0.67 (-1.05, 1.16)	1.56 (0.02, 5.77)	0.17 (-0.53, 1.91)	-0.08 (-0.75, 1.70)
[3]Lower Respiratory (summed Likert Score)	0.10 (-0.12, 0.69)	0.30 (-0.02, 1.08)	0.14 (-0.17, 0.99)	0.40 (-0.04, 1.50)	0.08 (-0.12, 0.48)	-0.08 (-0.24, 0.28)
[4]Mental Health (summed Likert Score)	1.18 (-0.38, 5.90)	0.06 (-0.96, 3.80)	0.58 (-0.98, 5.29)	1.23 (-0.69, 6.71)	1.00 (-0.38, 4.86)	-0.77 (-1.38, 1.78)
[5]Neurological (summed Likert Score)	0.42 (-0.44, 2.77)	0.53 (-0.41, 3.08)	-0.37 (-1.01, 1.66)	0.93 (-0.33, 4.12)	0.63 (-0.32, 2.83)	-1.04 (-1.38, 0.20)

[6]Gastrointestinal (summed Likert Score)	-0.06<br>(-0.18, 0.23)	0.10<br>(-0.08, 0.48)	-0.08<br>(-0.23, 0.24)	0.15<br>(-0.08, 0.60)	0.12<br>(-0.05, 0.42)	0.07<br>(-0.10, 0.40)
[7]Acute (summed Likert Score)	-0.07<br>(-0.63, 1.31)	0.99<br>(-0.02, 3.15)	-0.16<br>(-0.87, 1.57)	1.63<br>(0.20, 4.59)	0.31<br>(-0.32, 1.45)	-0.26<br>(-0.79, 0.76)

[a]Adjusted for age, sex, race, smoking, alcohol consumption, time spent in home, number of children <18 years in home, exercise, number of chronic health conditions, time of residence at current home, education level, and occupation. [2]Upper Respiratory = cough + nasal_congestion+ runny_nose + throat_irritation +nosebleeds. [3]Lower Respiratory = short_breath + lung_irritation. [4]Mental Health = anxiety_stress+ diff_sleeping + difficulty_concentrating + lack_energy_fatigue. [5]Neurological = dizziness + difficulty_concentrating + headaches + numbness_tingling + ringing_ears__hearing_loss + muscle_aches_weakness_pain. [6]Gastrointestinal = nausea + vomiting. [7]Acute = nausea + vomiting + Nosebleeds + lung_irritation + short_breath +cough + throat_irritation.

LCL = lower 95% confidence level, UCL = upper 95% confidence level.

Table S5. Difference in means for adult survey respondents living more than 2 miles, 1-2 miles, and < 1 mile from a multi-well oil and gas site in Broomfield Colorado, October -December 2021: Stratified by gender and chronic conditions[a]

	Identify as Male (N = 193)		Identify as Female (231)		Reported no Chronic Conditions (N=204)		Reported 1 or more Chronic Conditions (N= 223)	
Outcome	Difference between > 2 mile and 1-2 mile means (LCL, UCL)	Difference between > 2 mile and < 1 mile means (LCL, UCL)	Difference between > 2 mile and 1-2 mile means (LCL, UCL)	Difference between > 2mile and < 1 mile means (LCL, UCL)	Difference between > 2 mile and 1-2 mile means (LCL, UCL)	Difference between > 2 mile and < 1 mile means (LCL, UCL)	Difference between > 2 mile and 1-2 mile means (LCL, UCL)	Difference between > 2 mile and 1-2 mile means (LCL, UCL)
Total Number of Symptoms (N)	0.03 (-1.75, 1.81)	1.37 (-0.59, 3.33)	0.58 (-1.28, 2.41)	0.56 (-1.35, 2.46)	0.17 (-1.48, 1.82)	0.70 (-1.17, 2.57)	0.84 (-1.11, 2.78)	1.05 (-0.89, 2.99)
Total summed Likert Score	-0.03 (-2.34, 6,11)	3.12 (-0.75, 12.27)	1.16 (-1.67, 7.41)	1.03 (-1.82, 7.39)	0.71 (-2.03, 6.50)	1.44 (-1.89, 8.44)	1.29 (-2.17, 10.05)	2.55 (-1.43, 12.20)
[2]Upper Respiratory	-0.38 (-0.74, 1.15)	1.28 (0.02, 4.72)	0.23 (-0.52, 2.47)	0.62 (-0.34, 3.31)	0.19 (-0.51, 2.12)	1.26 (-0.01, 4.35)	-0.44 (-0.90, 1.60)	0.46 (-0.47, 3.46)
[3]Lower Respiratory	0.28 (-0.10, 1.09)	0.49 (0.02, 1.66)	-0.007 (-0.22, 0.53)	0.007 (-0.22, 0.57)	0.01 (-0.18, 0.39)	0.11 (-0.14, 0.57)	0.19 (0.13, 1.16)	0.33 (-0.06, 1.43)
[4]Mental Health	-0.07 (-0.66, 2.44)	0.44 (-0.49, 3.86)	1.88 (-0.05, 6.84)	0.01 (-1.05, 3.36)	0.95 (-0.41, 4.78)	-0.27 (-1.15, 2.83)	1.35 (-0.69, 7.76)	1.44 (-0.63, 7.94)
[5]Neurological	0.45 (-0.60, 3.53)	0.53 (-0.64, 3.98)	-0.002 (-0.60, 1.29)	0.29 (-0.47, 1.90)	-0.14 (-0.92, 1.90)	-0.35 (-1.14, 1.85)	0.56 (-0.43, 3.38)	0.41 (-0.50, 3.10)
[6]Gastrointestinal	-0.01 (-0.15, 0.28)	0.03 -0.14, 0.37)	-0.14 (-0.71, 1.52)	-0.06 (-0.69, 1.71)	-0.02 (-0.16, 0.25)	0.06 (-0.13, 0.39)	0.10 (-0.09, 0.50)	0.12 (-0.08, 0.53)
[7]Acute	0.12 (-0.57, 1.71)	1.21 (0.04, 3.68)	0.15 (-0.56, 1.67)	0.50 (-0.36, 2.26)	0.04 (-0.60, 1.25)	0.73 (-0.21, 2.42)	0.21 (-0.55, 2.08)	0.92 (-0.13, 3.27)

[a]Adjusted for age, sex, race, smoking, alcohol consumption, time spent in home, number of children <18 years in home, exercise, number of chronic health conditions, time of residence at current home, education level, and occupation. [2]Upper Respiratory = cough + nasal_congestion+ runny_nose + throat_irritation +nosebleeds. [3]Lower Respiratory = short_breath + lung_irritation. [4]Mental Health = anxiety_stress+ diff_sleeping + difficulty_concentrating + lack_energy_fatigue. [5]Neurological = dizziness + difficulty_concentrating + headaches + numbness_tingling + ringing_ears__hearing_loss + muscle_aches_weakness_pain. [6]Gastrointestinal = nausea + vomiting. [7]Acute = nausea + vomiting + Nosebleeds + lung_irritation + short_breath +cough + throat_irritation.
LCL = lower 95% confidence level, UCL = upper 95% confidence level.

Table S6. Difference in means for adult survey respondents living more than 2 miles, 1-2 miles, and < 1 mile from a multi-well oil and gas site in Broomfield Colorado, October -December 2021: Sensitivity Analysis for respondents identifying as white, never smoked or lived with a smoker, 55 years and older and lived in their home for 2 or more years.[a]

	Identify as White Alone (N = 387)		Never Smoked or Lived with a Someone that Smokes (N=361)		55 years and Older (N=249)		Lived in Home for 2 or more years (N= 223)	
Outcome	Difference between > 2 mile and 1-2 mile means (LCL, UCL)	Difference between > 2 mile and < 1 mile means (LCL, UCL)	Difference between > 2 mile and 1-2 mile means (LCL, LCL)	Difference between > 2 mile and < 1 mile means (LCL, UCL)	Difference between > 2 mile and 1-2 mile means (LCL, UCL)	Difference between > 2 mile and < 1 mile means (LCL, UCL)	Difference between > 2 mile and 1-2 mile means (LCL, UCL)	Difference between > 2 mile and 1-2 mile means (LCL, UCL)
Total Number of Symptoms (N)	0.34 (-0.95, 1.63)	0.96 (-0.42, 2.35)	-0.3 (-1.61, 1.01)	0.70 (-0.72, 2.11)	0.78 (-0.98, 2.54)	0.88 (-0.74, 2.45)	0.08 (-1.21, 1.36)	0.97 (-0.46, 2.40)
Total summed Likert Score	0.90 (-1.54, 5.40)	1.9 (-0.10, 7.18)	-0.67 (-2.57, 3.15)	1.64 (-1.16, 6.89)	1.17 (-0.91, 7.39)	1.34 (-0.68, 7.18)	0.08 (-1.97, 3.97)	2.36 (-0.61, 7.73)
[2]Upper Respiratory	-0.04 (-0.56, 1.20)	0.81 (-0.08, 2.70)	-0.48 (-0.88, 0.66)	0.75 (-0.19, 2.84)	0.13 (-0.25, 1.78)	0.49 (-0.07, 2.48)	-0.21 (-0.63, 0.87)	1.02 (0.06, 3.04)
[3]Lower Respiratory	0.11 (-0.10, 0.50)	0.16 (-0.07, 0.61)	0.003 (-0.18, 0.37)	0.22 (-0.05, 0.74)	0.04 (-0.10, 0.51)	0.07 (-0.08, 0.52)	0.10 (-0.11, 0.50)	0.17 (-0.08, 0.66)
[4]Mental Health	1.22 (-0.28, 4.57)	0.41 (-0.84, 3.41)	0.57 (-0.50, 3.10)	0.50 (-0.61, 3.17)	0.89 (-0.17, 4.99)	0.37 (-0.33, 3.41)	0.60 (-0.50, 3.11)	0.47 (-0.67, 3.15)
[5]Neurological	0.05 (-0.59, 1.45)	0.21 (-0.54, 1.80)	-0.25 (-0.75, 0.92)	0.21 (-0.50, 1.77)	0.38 (-0.31, 2.94)	0.20 (-0.34, 2.32)	-0.002 (-0.60, 1.29)	0.29 (-0.47, 1.90)
[6]Gastrointestinal	0.03 (-0.09, 0.23)	0.14 (-0.02, 0.39)	-0.05 (-0.13, 0.10)	0.03 (-0.08, 0.22)	0.01 (-0.13, 0.31)	0.06 (-0.08, 0.36)	0.008 (-0.11, 0.21)	0.06 (-0.09, 0.29)
[7]Acute	0.21 (-0.36, 1.18)	0.83 (0.05, 2.11)	-0.22 (-0.68, 0.63)	0.60 (-0.14, 1.86)	0.17 (-0.34, 1.55)	0.69 (-0.002, 2.33)	0.05 (-0.49, 1.02)	0.80 (-0.01, 2.18)

[a]Adjusted for age, sex, race, smoking, alcohol consumption, time spent in home, number of children <18 years in home, exercise, number of chronic health conditions, time of residence at current home, education level, and occupation. [2]Upper Respiratory = cough + nasal_congestion+ runny_nose + throat_irritation +nosebleeds. [3]Lower Respiratory = short_breath + lung_irritation. [4]Mental Health = anxiety_stress+ diff_sleeping + difficulty_concentrating + lack_energy_fatigue. [5]Neurological = dizziness + difficulty_concentrating + headaches + numbness_tingling + ringing_ears__hearing_loss + muscle_aches_weakness_pain. [6]Gastrointestinal = nausea + vomiting. [7]Acute = nausea + vomiting + Nosebleeds + lung_irritation + short_breath +cough + throat_irritation.
LCL = lower 95% confidence level, UCL = upper 95% confidence level.
